# Careful Co-presence: The Transnational Mediation of Emotional Intimacy

**DOI:** 10.1177/2056305119854222

**Published:** 2019-06-24

**Authors:** Donya Alinejad

**Affiliations:** Utrecht University, The Netherlands

**Keywords:** emotional affordances, social media practices, transnational migrants, careful co-presence

## Abstract

This article investigates how migrants experience “co-presence” with their loved ones through social media. On the basis of empirical investigation, the article engages with current debates about how social media shape emotional experiences. It draws on short-term ethnographic research of everyday social media practices among second-generation Turkish-Dutch migrants who grew up in the Netherlands and migrated to Istanbul in adulthood. The article focuses on transnational family intimacy within this migration phenomenon as an in-depth case study for understanding the role of social media platforms and mobile devices in producing emotional experiences of togetherness under conditions of long-distance, long-term separation. The author shows how social media platforms afford not only ambient, fast-paced, background communications—which have been emphasized in the literature, thus far—but also more direct, immersive, conversational modes of communication. The article argues that people’s practices of carefully shifting between these modes of social media communication produce their experiences of transnational emotional intimacy. The author develops the notion of *careful co-presence* through a discussion of how social media practices that produce intimacy reflect both discerning selectivity and emotional care. This argument builds on scholarship that has advanced practice-based approaches to understanding how emotion is mediated through digital media.

## Introduction

While conducting field research on transnational family relationships of second-generation Turkish-Dutch young adults, I was struck by how transnational everyday life had changed within these young people’s lifetimes because of developing technologies. Miriyam remembered how, as a child, she had made the move from Turkey to the Netherlands in her family car piled full of belongings. Similar to accounts of others I interviewed, she remembered how the first family trips visiting Turkey had also been in the car, before flights were affordable enough for the family to travel by plane. Miriyam described how family welcomes were a collective celebration, and goodbyes were emotionally intense rituals of crying, embracing, and passing everyone under the Koran for protection. “Because we wouldn’t see each other’s faces for months or years until the next trip!” she explained. “But nowadays everyone has Facetime and we fly over to visit, so there’s no need for such dramatic emotions.” Miriyam, and others whose lives had been characterized by living in transnational families from childhood, were particularly well positioned to offer insights into how emotional closeness was experienced in new ways as social media applications like Facetime proliferated into the everyday communications, helping to maintain family relationships across transnational distances. For researchers on transnational intimacy and care relations, this raises important questions about the changes and continuities that emerge in transnational emotional relationships with the rise of social media use, specifically.

There is a small but growing scholarship on how digital media forms are used to communicate and experience emotions in contexts of transnational mobility (Alinejad, 2011; Leurs, 2014; Madianou, 2016; Madianou & Miller, 2012; Nedelcu, 2012; Schrooten, 2012; Twigt, 2018; Wilding, 2006; Witteborn, 2014). This literature has touched on a range of different migration phenomena, approached from various disciplinary perspectives. Despite its diversity, one common theme across this work is the discussion of how migrants use digital media to cope with the distance from loved ones in the country of migration. This discussion has coalesced around mediated forms of intimate presence and, in particular, the concept of “co-presence,” which can be defined as an experience of emotional proximity with, or feeling close to, physically distant loved ones. While we know that transnational emotional relationships have long been mediated since letter writing and postal technologies (Thomas & Znaniecki, 1996), the proliferation of digital media into people’s everyday practices throws up the challenge of understanding the role of the latest iteration of Web 2.0 (also known as “the social web”) in emotional experiences of intimate togetherness. As Boccagni and Baldassar (2015) state of transnational families, “[n]ew media are transforming and creating new forms of co-presence and sociality that facilitate the constitution and maintenance of emotional bonds across distance and over time” (p. 75). And longitudinal research has shown that people’s ability to feel co-present has grown over time with new technologies that afford synchronicity becoming available (Baldassar, Nedelcu, Merla, & Wilding, 2016). Web communications, thus, seem to be changing how migrants feel close to their loved ones abroad.

Scholars have identified various kinds of digitally mediated co-presence. “Ambient co-presence” was proposed by Madianou (2016) to discuss how transnational families constantly have the feeling of each other’s presence through the “‘always on’ culture of ubiquitous connectivity” (p. 198).^1^ “Ordinary co-presence” is the term Nedelcu and Wyss (2016) use to discuss how transnationally mediated communications mirror “normal” family functioning and are an expression of cosmopolitan everyday life. These and other scholars of migrant digital media practices (Diminescu, 2008; Wilding, 2006) have been inspired by Christian Licoppe’s notion of “connected presence,” which argues that because of a “changing technoscape” shaped by mobile phone calls, text messaging, and emails, “the boundaries between absence and presence eventually get blurred” (Licoppe, 2004, p. 136).^2^ Those drawing on Licoppe’s “connected” modes of communication to discuss transnational migrant intimacy tend to emphasize how digital media (and social media platforms, in particular) afford a continuous, ephemeral, and constantly available mode of communication. For instance, Diminescu (2008) states that “connected” modes of relationship-maintenance through digital media have transformed presence into more of an affective experience than a physical state, and thus “revolutionized” migratory practices and experiences of mobility. However, Licoppe’s original analysis holds that both older forms of “conversational” communication and newer forms of “connected” media repertoires, though distinct, continue to exist alongside one another. Furthermore, Licoppe (2004) concludes that the fleeting communicative acts that define “connected” communication do not necessarily produce feelings of a strong intimate relationship; this requires an effortful intensification of communication, producing “a common world of signification” (p. 154). Put simply, constant digital communication need not produce intimacy, just as not all silences signify emotional distance.

For instance, Madianou (2016) points to moral surveillance as a downside to intense transnational co-presence, and mentions that certain digital forms can help to purposefully introduce distance into an intimate relationship. In addition, Madianou and Miller (2012) describe situations in which transnational familial love is sometimes expressed more readily through digital media than face-to-face, suggesting that “mediation can potentially create the ‘ideal distance’ necessary for a relationship to flourish” (p. 146). Nedelcu and Wyss (2016) further develop a discussion of purposeful distance through their notion of “ordinary co-presence,” examining how modern family life is inherently constituted through distance and difference, and they explicitly challenge the idea that communicative distances or silences reflect emotional disconnection per se.^3^ This suggests that transnational intimate relationships may to some extent rely on silences, omissions, and absence, and involve a variety of communicative needs and strategies. Thus, the experience of transnational family intimacy is mediated in more complex ways than we can understand through a focus on how constant, synchronous communication leads to people feeling close despite physical distance. While migration studies research has compellingly shown that transnational family relationships of care and intimacy span distances and circulate with human mobility (Baldassar & Merla, 2013), we know far less about how social media shape the ways people experience the emotional dimensions of their distant and mobile care relations.

In this article, I investigate transnational migrant co-presence primarily as an emotional experience. I ask how this experience is mediated in the age of the social web, through social media apps and platforms, specifically in situations in which everyday intimate lives are lived across transnational distances. I show how the mediation of intimacy takes shape around particular social media affordances that emerge as a part of a mode of deliberative and emotionally constitutive social media usage I refer to as *careful*. I use the *careful* descriptor in reference to the social media practices that I argue produce experiences of transnational co-presence. These are *care-full* in the sense of involving feelings of care as discussed in the scholarship on how care circulates dynamically within transnational families (Baldassar & Merla, 2013), while also involving *careful* and discerning selection between different available media practices for staying in touch with loved ones within economies of time and attention in the digital age (Terranova, 2012). Conceptualizing the integration of carefully selected media practices and practices of transnational care, c*areful co-presence* is inspired by the notion of “emotional affordances” put forward by Bareither (forthcoming), which theorizes emotion and digital media affordances from a practice-oriented, ethnographic perspective that draws from the anthropology of emotions to argue that digital media change emotional experiences by shaping the (bodily) practices through which emotions are enacted. In what follows, I demonstrate how the idea of careful practices of producing transnational intimacy through social media emerged from my empirical material. First, I discuss how direct messaging takes on new significance in the context of contemporary feed-scrolling social media, revealing how control over what is shared with whom may be a way of preserving intimate relationships. Then, I show how economies of time, focus, and availability produce specific digital media routines that produce transnational intimacy within the physical spaces of the home. And finally, I elaborate on how social media offer ways to stake out emotional independence from family members. After which I conclude by pointing out some of the further theoretical implications of my discussion of the empirical material. But first, I reflect on the methodological approach I used to collect the empirical data.

## Short-Term Digital Ethnography

In order to investigate how social media mediate co-presence or a feeling of togetherness and emotional proximity, I focused on a specific set of users. These were second-generation Turkish-Dutch migrants who decided in their adulthoods to move from the Netherlands (where they lived significant periods of their young lives) to Istanbul, Turkey. This was one of the groups that are the focus of an ongoing research project on migrants and digital communications across the shifting borders of Europe.^4^ There is little research on emotional experiences of the Turkish-Dutch second generation, and the few studies on emotion are focused on cultural belonging in the Netherlands (see Arends-Tnds and van de Vijver, 2004) rather than transnational relationships or those who migrated (back) to Turkey. The study of emotions within this group is overshadowed by research focused on their cultural, educational, and labor market integration in the Netherlands, an interest that reflects the Netherlands’ historical relationship with Turkey. Turkish migrants to the Netherlands became the largest foreign labor population in the country in the 1970s (AkggNeth, 1993),^5^ and have since become the group with the highest “return” migration rate from the Netherlands.^6^ In fact, migration between Turkey and the Netherlands has entered a phase in which the overall historical flow has been reversed, with more emigration to Turkey than immigration from Turkey.^7^ At the same time, the children (and grandchildren) of this cohort of migrants to the Netherlands—also referred to as second- (and third-) generation Turkish (-Dutch) migrants—have begun entering adulthood in the country. They have come to comprise more than half the Turkish-Dutch population.^8^

In recent decades they have attracted a notable amount of scholarly, policy, and media attention in the Netherlands regarding their economic and educational participation, but also with respect to contentions around their apparent loyalties toward the Netherlands and Turkey. Within the past 5 years, second-generation Turkish-Dutch young people emigrating to establish their lives in Turkey have become a significant portion of those emigrating to Turkey from the Netherlands.^9^ And although those leaving are a minority of the wider second-generation population,^10^ they constitute both the largest (absolute) number and the highest proportion of second-generation emigrants from the Netherlands.^11^ Among my respondents, the motivations for this move mostly included professional opportunities in a country where their bilingual/bicultural backgrounds are an asset, a cosmopolitan urban life in Istanbul, and/or past experiences of discrimination in the Dutch job market. However, those who had moved with hopes for Turkish economic growth being accompanied by an opening up of Turkish creative and civic society had grown increasingly despondent about this possibility. This led to a number of my respondents looking into moving back to the Netherlands around the time I was conducting this research.

In this article, I present a discussion of the interview and observation material from 4 months of “short-term ethnographic” field research (Pink & Morgan, 2013) I carried out in Istanbul in 2017, which was focused on respondents’ everyday social media platform and mobile device usage practices. The research approach comprised in-depth, semi-structured interviews with 15 second-generation Turkish-Dutch respondents living in Istanbul, some of whom were interviewed repeatedly. I also conducted informal interviews with four second-generation Turkish-Dutch young people living in Amsterdam (making a total of 19 interviewees). These offered points of entry for snowball sampling via their Turkish-Dutch peer contacts living in Istanbul, while the remainder of the respondents were selected through theoretical sampling (Howard, 2002). They had either traveled extensively to Istanbul in their adulthoods, or they had moved there before deciding to move back to the Netherlands. The respondents represented a skilled, upwardly mobile group with middle-class socio-economic status, aged between their twenties and forties; 11 of them were men and 8 were women. The fieldwork involved attending social gatherings, home visits, and neighborhood walks with my respondents in the areas of the cities where they lived in Istanbul.

## Direct Messaging as a Site of Transnational Intimacy

Sinan was a young man in his early thirties whose parents were originally from Bodrum, Turkey, but had moved to the Netherlands before his birth. Having grown up and gone to school in the Netherlands, while having made habitual summer trips to Bodrum and Istanbul for family visits and tourism, Sinan decided he wanted to move to Turkey. He narrated his move to Istanbul as a key decision in a period of his life when, much like many of my other respondents, he was generally preoccupied with developing himself personally and professionally. He also wanted to gain some independence from his immediate family and his living environment in The Hague in the Netherlands. Sinan had moved to Istanbul a few years prior with the intent of settling his life in the city in the foreseeable future. He noted that Instagram was particularly popular in Istanbul, compared to in the Netherlands—something I had also observed in my time in the city. And yet, Sinan said he was not so active on the platform. He explained:

The reason I don’t share a lot on Instagram is because my mother started following me there. And on Facebook too—at some point she had kind of discovered them. My parents would prefer me to live differently. If I’m sitting somewhere with a beer then I would rather not share that. And yeah, then your father or your mother sees that and, yeah it’s just not cool. And then you don’t have that much to post, because I am someone who is in the bar really often. So that was actually the reason. For them it was really great at the beginning—I would post photos all the time. But then they saw this photo with girls. And they didn’t like that, because then they knew that I was doing those kinds of things. And one time I had a bit of a fight with them, and then I blocked them on Facebook. I blocked my parents on Facebook [laughter]. On Instagram, not yet. But I just post less there anyway. . . My family is more religious. My parents would be like, look, look what he’s doing there. And then he even went and shared it! [laughter]^12^

Sinan’s choice to first add, and then block, his parents on Facebook made me interested in how he negotiated his social media contact and relationship with his parents in the Netherlands, particularly within the social environments of Facebook and Instagram that my respondents typically used to mediate peer sociality. Sinan told me he had decided to limit his contact with his parents to WhatsApp, a direct messaging platform. Indeed, for communications within their intimate, long-distance relationships, most of my respondents used WhatsApp, Facetime, and other messaging possibilities of other apps—platforms that primarily foster direct, one-to-one communication. They chose these over social media platforms that are defined by their feed-scrolling formats. In fact, most of my respondents did not immediately see WhatsApp as a social media platform, at all. In Sinan’s case, direct messaging afforded a higher degree of control over what was communicated with whom than Instagram or Facebook, which was why he limited all contact with his parents to WhatsApp. But as part of his continued negotiation of the boundaries of sharing his Istanbul life with his family, Sinan eventually updated his WhatsApp profile photo to a portrait of himself holding a beer and a smartphone. Wondering what had led to him sharing this side of himself that had caused problems with his parents before, I asked about the profile photo. “I don’t let it bother me as much as it used to,” Sinan replied. I saw this as a part of the way he lived his preferred lifestyle in Istanbul while selectively sharing and concealing elements of it with his parents in shifting ways over time. I saw WhatsApp as having made it easier for Sinan to carefully manage this moving boundary through the control it afforded him, especially compared to updates and photo-sharing on social media feed formats, with their relative a-synchronicity and their wider and more amorphous scales of privacy and sociality.

Sinan was born and lived for 28 years in an underserved neighborhood of The Hague, the Schilderswijk, a historically working-class area that later became notorious on a nation scale in the Netherlands for urban degradation, street crime, and its concentration of residents with Turkish, Morrocan, and other migrant backgrounds. Sinan said:

I lived with my parents for a long time. I kind of just couldn’t get away. And my parents had this idea about it like: you should get married and otherwise you shouldn’t move out. And at some point I got sick of it and just came here. Not only for that reason, but it had been a dream of mine, to come to Istanbul . . . I came about 17 or 18 times to Istanbul before I actually moved here. So I really loved Istanbul.

Sinan told me about having been caught up in a drug-using peer environment in The Hague, and had been concerned about what would happen if he had moved out alone without breaking with that. He also described experiences he and his friends had with ethnic profiling by police in The Hague, which led him to move around that city with more trepidation. He had developed an emotional bond with Istanbul since the summer family trips of his childhood, and his parents had bought an apartment there some years back, where other members of their extended family in Istanbul had been living for a while. Once the apartment was free, Sinan bought a ticket to Istanbul to see how life would go in this partly new, partly familiar place. His stories reflected his navigation across the social worlds of his fraught but familiar neighborhood life in The Hague, his relatively conservative Turkish family life in the Schilderswiik, and his progressive Istanbul nightlife. And it was through these social crossings that Sinan’s social media usage called for a careful management of emotional distance and proximity to the people, places, and social life he had left behind.

Migration studies research on emotions in mediated transnational family relationships previously emphasized how physical presence was the “gold standard” of experiencing co-presence, against which “new forms of co-presence” are posited as lesser experiences due to their being mediated (Baldassar et al., 2016). With her notion of “ambient co-presence,” Madianou re-explicated the idea that digitally mediated communications are no less significant simply because they are indirect and in the background of everyday life, and argued that ambient co-presence “enhances users’ sense of belonging by immersing them into emotional and moral spaces” (Madianou, 2016). In Sinan’s case, the distance that migration introduced allowed him a fresh start outside the social peer circles and conservative family life he had felt as a pressure. For his relationship with his parents, his physical presence in their home brought with it certain emotional tensions, and these extended initially into his Istanbul life via social media when his mother could see his Instagram profile. Hence, as Baldassar has also admitted, drawing on Madianou and Miller’s (2012) influential work, distance is not necessarily the source of tensions in long-distance family relationships (Baldassar, 2016). For Sinan, the distance that migration put between him and his parents was an intentional choice, as was his eventual move to share selected aspects of his life with them through WhatsApp. It is this careful balancing of distance and emotional proximity that seems to characterize Sinan’s practices of shifting between platforms and choosing a way to control and direct his communication.

Affective and emotional dimensions of social media are often conceptualized in terms of the always-on, omnipresent, ambient, “connected,” and rapid-paced affordances of social media platforms, in combination with the mobile devices on which they are typically used (Madianou, 2016; Papacharissi, 2016; Wilding, 2006). In accordance with this, emotional affordances of social media are conceptualized in terms of a more peripheral and “lightweight awareness of connection with others through the online space” (Madianou, 2016), or a lack of reliance on the direct and more immersive interaction of the phone call. Indeed, my respondents talked about how such social media affordances shaped how they communicated news and major life events between themselves and their family members. However, they also mentioned how these modes of communication distracted them from intimacy, and how these fast-paced, indirect, and brief forms of communication failed to maintain their close emotional relationships. In the context of social media ubiquity and ambient communications, the choice for direct communication seemed to take on new significance for people. For example, Umut, another young man in his thirties who worked for an event-planning agency, had moved to Istanbul for work. Umut said he only used Facetime and Skype to make video calls to his family in the Netherlands, which I discuss further in the next section of this article. He took up letter writing at a certain moment to communicate with his former girlfriend in the Netherlands, saying:

In the first place, it’s more personal. And when you write you can say more because you really think about what you’re going to say. A message is fast. You don’t always think about what you’re saying . . . [letter writing] is more romantic . . . there’s a sort of nostalgic feeling about it as well.

Umut even said he sometimes wished he had a girlfriend he could still write to. This focus on direct communication, whether through voice calls, video calls, or in Umut’s case, even letters, seemed to take on an important role in relation to the ephemerality of other social media communications. Most of my respondents said they stayed in touch with their parents in the Netherlands through WhatsApp, which had taken the place of conventional telephony. They used WhatsApp for (video) voice calls as well as texting, such that phone calls remained important, and some gave accounts of helping their parents switch to calls via WhatsApp so that they could use Internet telephony. In some cases, the long-distance calls were the sole reason their parents used such apps. Huseyn, a young man who moved from the Netherlands to work for the Turkish branch of a Dutch bank in Istanbul, explained that this had been the case for his mother:

The thing is, when I first moved to Istanbul [in 2012], social media wasn’t being used as it is today. I’m quite lucky that I moved around that time because before 2012 it was less attractive—there were less new things. So the first thing my mom did was buy a smartphone when I moved here. And downloading all the apps like WhatsApp and [. . .]also Facetime, Viber. [. . .]. She downloaded these apps for herself to stay in touch with me. It was good timing, because if it was in 2007 or 2005 I think we would have had a huge invoice to pay. So it was easy to get in touch because we used those apps.

Most people described using WhatsApp or another voice calling app to communicate with their parents on a regular basis. This was their main mode of communicating with close family members left behind, whereas they mostly used short WhatsApp messaging to stay in touch with people they saw locally on a day-to-day basis. In the context of the feed-scrolling format that is seen as the main characteristic of social media platforms such as Facebook, Instagram, and Twitter, these young people use direct forms of communication as ways to control the content of what they share with their parents. As Sinan’s case reflected, they use this affordance to create separated social spaces. This relates to debates about the concept of “context collapse” (Marwick & boyd, 2011), in which some have influentially argued that the collapse of different social contexts onto one another is a platform affordance of social media, one which is consequently limited through active measures for “strategic audience management” (p. 128). However, in her study of Facebook in the medium-sized Turkish border city of Mardin, Costa argues from a digital anthropological perspective that an affordance is not a mere property of a platform, but only becomes apparent through media usage in various different social and cultural settings (Costa, 2018). She builds this on her finding that the idea of context collapse fails to account for how her Turkish respondents use Facebook, namely, consistently utilizing complex privacy settings to reproduce local social norms of keeping various social circles separate from one another. In this case, beyond creating a clear separation between distinct social worlds according to social norms, the choice of direct, one-to-one communication seems to reveal the affordances of calling and direct messaging apps for maintaining the close relationships that are specifically emotionally intimate. This is done in ways that emulate and augment phone calls and letter writing more than they do away with these older forms of communication. The ways affordances have been discussed in the debate around context collapse seems to underemphasize the *emotional* dynamics of managing relationships through social media in favor of either local cultural norms concerning social relationships or generic platform capabilities. I elaborate on the emotional affordances of these direct communication practices further in the following section, where I discuss how people use messaging apps within everyday routines and places.

## Media Routines and Home Spaces

Something my respondents almost invariably associated with their everyday social media use in Istanbul was the tendency to get WhatsApp messages from colleagues, including outside work hours. Overall, long working hours, distant commutes, and busy socializing schedules overlapped with being collegiate within and outside the workplace, leaving few openings for transnationally “doing family” online (Nedelcu & Wyss, 2016). This time pressure was reflected in people’s app usage and the way they managed their social media use so as to avoid becoming overwhelmed. Some typically used apps that supported a lifestyle of fast-paced, urban mobility and convenience, such as the extrEsray popular Yemeksepeti food delivery app (which a number of my respondents swore by) and the Yandex traffic app, both of which were oriented toward local usage and saved time by helping to avoid traversing long, congested city routes.^13^ While some people reported deleting certain social media apps so that they could avoid spending too much time on their smartphones, others did this by personalizing apps to only draw their attention with notifications about the things they cared about most. For instance, Huseyn stayed up-to-date with news from the Netherlands (both about the Netherlands and about Turkey) via the Dutch news app, NOS. He set it to notify him only on topics he was interested in: “global finance, economy, Turkish-Dutch relations, that kind of thing, and apart from that not so much.” He had become very careful about becoming overloaded in this way. He had strained his hand from using his smartphone too much, and wore a wrist brace when I met him. “Nowadays they call it telephone hand,” he said.

He was not the only one who had taken steps to reduce and be extrEsray selective about their smartphone usage. Sinan said that he had decided to delete the mobile Facebook app as a way to avoid going on Facebook too much throughout the day. Umut said he had closed his Facebook account, mentioning that he used Instagram instead because it took less time to stay up-to-date because you “don’t have to read the long updates people write”—he lamented that in contrast with Instagram, “you can spend hours on Facebook.” Hakan, a young man who had moved to Istanbul to work in the design sector, explained that he consciously used Facebook less since his Instagram activity was already quite intensive. He only kept his Facebook account open because of the contacts whose phone numbers he did not have, and the collection of photos he would lose. Many saw themselves as avoiding Facebook because it took up too much time in their day.^14^ Their everyday lives in the megacity of Istanbul as skilled, early career professionals appeared to require careful management of app usage. I see these local daily circumstances as giving meaning to practices of allotting time for more extended phone calls and direct communication without other distractions. Given my respondents’ middle-class background, this practice might also be seen as part of a wider shift that has been recently argued that Facebook is undergoing in its domestication process in middle-class user environments, a stage characterized by fewer emotional affordances and more mundane uses such as “scheduling,” and “archiving” (Sujon, Viney, & Toker-Turnalar, 2018). This may be a way in which new media forms change the meaning of, or augment the affordances of, older media forms (Bolter & Grusin, 2000), or how multiple platforms used in parallel take on meaning with relation to one another as polymedia (Madianou & Miller, 2012).

In Umut’s case, his closest relationships with family in the Netherlands were with his cousins, and these required periodical moments in which he used Facetime to make voice calls from the furnished rental apartment in which he lived alone. There, he would have time to talk without other people around. For Umut, like others whose homes I visited, a typical mode of using his smartphone in the living room took place on a weekday evening, sitting on the couch in front of the television with his work and private smartphone in hand or on the couch next to him, connected to the charger cable. Others I interviewed at home also had routines of smartphone use oriented around the locus of a specific position on the living room couch. The physical surroundings in which the apps were used, and the daily routine of charging the digital device while not mobile throughout the bustling city, seemed to produce the conditions for intimate communication experiences that transient, mobile messaging throughout the course of the day did not provide. Umut describes his relationships with his cousins in the Netherlands as an escape from the hectic life he has in Istanbul. Although he does not see them often, he describes their bond having developed into an especially close one over the 20 or so years they have known each other, “so I can talk about everything,” he says. However, he decidedly did not think of them as a part of his social life in Istanbul. For him, that was an important part of the appeal of the transnational relationships they had. With his cousins, Umut explained, he could be the same person as before he left the Netherlands:

I still talk the same with them like I talked to them when I was there. Nothing’s changed. So, they’re not a part at all of my social life in Istanbul. For me, they’re a kind of escape from my social life in Istanbul. I go back to basics.

Umut’s Facetime calls with his cousins appear to produce pleasant associations with earlier times of togetherness and associations of family life in the Netherlands before his migration to Turkey. The calls evoke a distant time, place, and identity for Umut, and this distance allows him to experience an emotional release from the pressures of his daily Istanbul life. This practice does not threaten to collapse his long-distance relationships into the social context of Istanbul. On the contrary, it is mediated through immersive and undistracted voice calls that also separate him from the distractions of his Istanbul setting. However, in Umut’s case, the reason for choosing this mode of communication is not to strategically maintain separation between social spaces in accordance with social norms, but because a less immersive media form would fail to mediate the proper emotional intimacy to allow him an escape from his demanding life. As in Sinan’s example discussed in the previous section, Umut’s separation of different social spaces on social media is a side effect of choosing narrow channels for communication with loved ones in accordance with the different needs for emotional closeness or distance. He also contrasts the calm escape of phone calls with loved ones to the anxiety he experiences on the evenings he spends reading the Turkish language news on Twitter (from a few journalist’s accounts he trusts) about the domestic economy and Turkey’s international relations situation.

The media routines discussed in this section additionally reveal the immersive experience of voice calling. This appears to be especially meaningful in an urban (media) environment of intensely competing demands on people’s attention through constant prompts, and is perhaps particularly true of life in a megacity like Istanbul,^15^ where a growing awareness of the downsides of intensive social media use is catching on as being part of a tech-savvy lifestyle. This reflects the condition boyd describes, in which social media platforms embed their users in an “attention economy,” where attention is a limited resource (boyd, 2010). This idea has been elaborated to relate to emotions with the concept of “affective publics,” albeit with a focus on public emotions in the context of political participation (Papacharissi, 2016). Concerning emotional communications in close personal relationships, others have discussed how people’s contemporary notions of privacy and intimacy are produced by creating a contrast with the “affective qualities” associated with platforms like Facebook (McVeigh-Schultz & Baym, 2015). This suggests that some social media platforms afford intimacy through the escape or contrast they offer from engaging in media worlds of distraction.^16^

In my research, Facebook is particularly identified as contributing to distractive modes of media use, and I see this as having implications for the platform’s potential for mediating intimate emotion. For instance, one young woman, Meyra, who was settled in the Cihangir neighborhood of the city along with a group of peers who also had Turkish-Dutch backgrounds, said that in contrast to WhatsApp, which she uses intensively, “Facebook is more for the people you are not so close with, I would say.” Looking closely at the media affordances that emerge in these intimate communications reveals the important emotional facets of everyday social media use under the local circumstances. These are specific to a context where economic anxieties were omnipresent during this period in Turkey, including in various media spaces. But they are also anxieties produced by neoliberal structures that create demanding everyday life circumstances in cities, which are perhaps similar to other advanced urban societies (Çaglar & Schiller, 2018) in which social media ubiquity is experienced as promoting distraction from intimacy.

## Migration and Negotiating Emotional Independence

Huseyn told me that he calls or sends text messages to his mother regularly, but is very aware that his sisters and mother are in touch with each other far more frequently. His two sisters and younger brother live in Eindhoven, the same small city where his parents live, and where he also grew up. Since his sisters moved out, they are very actively involved in their parents’ lives on a day-to-day basis. But Huseyn did not see his sisters’ closeness to their parents as simply a product of their physical proximity alone. “They talk all the time. I don’t have contact with them every day, though. I love my mother and all, but I’m a man,” he says with a slight smile. “I also don’t speak with my friends every day either. They don’t need to call me unless there’s something going on. Or maybe in the weekend if we do something together.” In contrast, Merel was casual and open about the fact that her mother, who lives in Amsterdam, would message her every day. Merel was the child of a Dutch mother and a Turkish-Dutch father. Her friend, Meyra, noted that when she had first moved to Istanbul and been very busy, she had not had the time for long-distance phone conversations, relying instead on WhatsApp messaging. But now she was used to frequent calls to/from the Netherlands supplementing the messages. While the women I interviewed were also living independently from their families, there seemed to be slightly different stakes for young men in positioning themselves as independent from their families after their migration.

When Faruk had first moved to Istanbul and was getting settled, he had failed to stay in touch with the one of his two sisters with whom he had the closest relationship in the family. She had felt hurt by his silence, which he found out after some time from his mother—his sister had shared her disappointment in Faruk with her. Upon hearing of his sister’s upset, Faruk called her to resolve the issue, explaining that his silence was because he had expected her to get in touch with him, instead. After this resolution, which took place via phone calls, the atmosphere was better between them. But Faruk noted about himself that he was generally more stoic in his manner than his other family members were about his distance from them. “I don’t usually let myself get carried away by emotions,” he said. “Of course I miss them, but I see it [the distance] as *part of life*.”^17^ Faruk had chosen to move to Istanbul after he completed his Bachelor’s degree to continue his studies in International Business, and said that after migrating he felt he had finally “begun living.” It was the first time he had moved into his own place. After he had spent the initial months staying at his uncle and aunt’s apartment on the Asian side of the city, he had found himself an apartment on the European side and felt he had gained his independence, while still having his cousins to spend his free time with. For many of my respondents, mobility to Istanbul signified a similar, refreshing new stage of life, one they knew would involve perseverance and competition, but which they also felt would lead to greater independence and opportunity. The distance from family was often something they adjusted to readily as a part of this tradeoff, but they also found themselves helping their family members adjust to being apart from them. In Faruk’s case, this meant that he gradually convinced his parents to be supportive of his move by showing that they need not worry about him by managing to find a job, enrolling in a program at Bogazici University, and finding a rental apartment.

Even those who were slightly older than Faruk experienced the responsibility of alleviating parental worry from afar. And for some, this was a reason for being less communicative with their family members in periods when everyday life was more difficult. I especially heard this from men. Both Umut and Huseyn said they chose not to talk about their difficulties with their parents in order to avoid worrying them. Huseyn says that he tries not to worry his mother because he knows that the distance makes it harder for her since she is not able to do anything to help in most situations, anyway. Hence, he prefers not to upset her, unnecessarily. By contrast, when he would speak to his cousins, Umut was not always as open when speaking with his parents:

Umut: When I speak with my parents, they ask me how I’m doing and if I need anything. But I don’t always tell them if things aren’t going well. I won’t do that. Because I know them, I know they aren’t going to be okay.

Interviewer: Like, worried?

Umut: Yeah. Thinking about me all the time.

The feeling of closeness to family appears to be moderated here by the need to perform emotional independence from parents as a young adult. Hence, for some, the choice to migrate brings with it the responsibility of reassuring parents about one’s social, economic, and emotional wellbeing. And sometimes the best way to do this is to keep certain difficulties to oneself. Overall, my respondents had varying degrees of independence from their parents. Sinan lived in the furnished apartment his parents had bought as an investment some years back. And Esra, a young woman working as an editor, showed me the dining table that she had inherited from a grandfather who used to live in Istanbul, and the Philips coffee machine she had taken with her from her parents’ house in the Netherlands. And Meyra told me about how her parents had helped her bring new furnishings for her house over from the Netherlands. My respondents’ parents helped them to different degrees to feel supported and settled in Istanbul, and these young people seemed to feel the need to build largely self-sufficient lives for themselves in a combination of efforts to reciprocate parental care and become less reliant on it. As they receive the emotional care of their families from a distance and manage their emotional reactions, they perform success and stability for their parents through their social media communications. For some, the emotional intensities of family life are managed more effectively from Istanbul. Cengiz, for instance, described an emotional culture that was shared among the Turkish families he grew up among in the Netherlands in the 1990s. He recounted his earlier struggles with his overly emotionally reactive home environment, which was something he found he had in common with other second-generation Turkish-Dutch young people:

In the end, you do share a lot of things. Because we grow up in Turkish families in the Netherlands, so we all have these more autocratic fathers and how do you say, pastry-baking mothers. . . [W]hen I first went to my Dutch friends’ homes in primary school I was really surprised that their parents would not get angry if I would spill milk on the table or on the floor . . .

Cengiz saw highly emotionally charged relationships and encounters in the home as a common feature among the Turkish-Dutch families in his environment. This was something he associated with the Turkish-ness of these families, pointing out that it could lead to too much negative emotional intensity, sometimes. But it was equally something he related to positively at other times, as he also associated Turkish-ness more with “showing affection, touching, sharing.” Describing the way he saw himself he said, “my way of thinking is Dutch, my way of feeling is Turkish,” and,

I’m not living here because I feel so Turkish. I do live here because I like the fact that people here are more emotional. And the positive emotions I mean, the emotions I like. So they show their affection more, they’re more touching, they’re more open, they’re more sharing. I like that much more, there’s much more room for emotions. In the NL when you show the basic emotions, people say, oh you’re so emotional . . . I really feel, like, in between.

For Cengiz, moving to Istanbul had helped him feel more in touch with a way of being Turkish that placed some distance between his own Turkish-ness and the Turkish community of families he grew up within in the Netherlands. Some of my other respondents also mentioned their identification and/or dis-identification with Turkish-ness in terms of strong expressions of emotions they saw as culturally idiosyncratic, either in positive or negative ways, often contrasting this mode of emotionality with (their) cultural Dutch-ness, as Cengiz did. I saw this emotional discourse about differing Turkish and Dutch national cultures as expressing how far my respondents felt they fit in with, or alienated from, Turkish-ness or Dutch-ness. Living in Istanbul allowed them to live these feelings in different ways than living in the Netherlands had, allowing them to associate Turkish-ness with people beyond their families, circles of family friends, and neighbors in the Netherlands who were described by some as relatively socially conservative, working-class, and from provincial backgrounds. The Turkish-ness many discovered in Istanbul, in contrast, was more urban, diverse, cosmopolitan, and relatively open-minded, values these young people cultivated in their lifestyles in the city, and more specifically in the relatively small set of neighborhoods their worked, lived, and socialized within. In this context, I saw people’s social media use within their transnational families as a complex articulation of concerns about personal independence and their changing relationships with siblings and parents in this particular stage of young adulthood, as well as an invested care for their family members by limiting contagion or transmission of negative emotions transnationally. As I have tried to show, these communications are fumbling and based on trial and error while also crossing national and local cultural contexts, reflecting how social media practices are taken up as a part of ongoing and dynamic negotiations of transnationally mobile, second-generation migrant subject formation. Practices around transnational video and voice calls with family members in the Netherlands in this particular migration setting reflect users’ layered modes of reproducing and transgressing the (gendered sibling–sibling, parent–child, cross-/intercultural) relationship norms that govern participation in family life while articulating their independent, urban, subjectivities through these mediated negotiations of their transnational relationships.

## Conclusion

In transnational family relationships, sharing intimacy from a distance relies increasingly upon the ways communications technologies allow new forms of co-presence in a person’s absence. We know that in contemporary “polymedia” environments, where various different options for communication are available, transnational family members choose between social media platforms and the different possibilities and restrictions they offer for experiencing intimacy with others (Madianou & Miller, 2012). Influential scholarship on transnational co-presence has argued that the ephemeral, continuous, and ambient qualities of digital communications are changing transnational social relationships in line with the device and platform affordances of “always-on” social media apps and mobile smartphones. In my study of social media uses among second-generation Turkish-Dutch migrants to Istanbul, I focused on how these young people maintain transnational relationships with their family members with Turkish backgrounds in the Netherlands. I observed a strong preference for apps oriented toward intensive direct messaging and voice and video calls (in some cases, combined with a preference for being statically positioned in a usual spot within the private space of the home in order to avoid distraction). This indicated a sustained reliance on the affordances of relatively older formats of telecommunication such as sms text messaging and phone calls for mediating intimate relationships in the age of the social web, while also revealing how these affordances appear to take on additional significance in the context of social media ubiquity and urban professional life’s many demands on attention. These uses revealed somewhat opposing but parallel platform affordances of ambient, indirect, constant background communication, on one hand, and direct, immersive, conversational affordances, on the other. Showing how older and newer modes of media practice exist in parallel complicates certain uses of the “connected” descriptor in the current literature on migrant co-presence that tend to understate present-past media continuities and overstate transformation.

Paying particular attention to the emotional dimensions of these communications, I have argued that rather than a particular social media affordance transforming the experience of transnational intimacy, the *particular mode of communicating* with family through social media is what produces experiences of intimacy. I have called this a *careful* mode of communication, which produces experiences of mediated co-presence and distance between Turkey and the Netherlands in these relationships. I mean “careful” in the sense of both involving care and sensitivity, as well as being choosy and cautious of mistakes; it involves selection of where and when to communicate, as well as expressing emotional care for those being communicated with. I contend that this *careful* mode of social media practice both produces and conveys intimate emotional experiences of intimacy, an idea inspired by recent work that draws on practice theories of emotion and digital media to argue that emotional experiences are produced by media practices while at once also being expressed and performed through those media practices (Bareither, 2017). I also draw on theories of mediation that see media as not merely subject to use by people but also always training certain sensibilities through people’s media engagement (Grasseni & Gieser, 2019). Following from this approach to digital media forms and their “emotional affordances” (Bareither, forthcoming), it is important to address how people practice their competencies of selectively combining, managing, and shifting between different social media uses as an integral part of their situated emotional relationships. I have aimed to show how this is especially important to understand in contexts of transnational migration, where intimacy is mediated over sustained periods of distance, but also where mobility means crossing socio-cultural contexts in which social media platforms are used.

## Notes

1. The notion of “ambient virtual co-presence” first appeared in the digital media scholarship on intimate relationships under conditions of mobility more broadly (Itō, Okabe, & Matsuda, 2005).

2. There is a growing scholarship investigating personal, intimate, and emotional facets of social media’s role in social life, especially how it shapes human relationships (Baym, 2010; Chambers, 2013, 2017; Garde-Hansen & Gorton, 2013; Kraemer, 2014; van Dijck, 2013). For the purposes of this article, I limit my engagement with the literature to the work that has focused on digital media use in contexts where transnational migrants’ relationships, in particular, are being mediated.

3. See also Madianou and Miller (2012) on “ideal distance.”

4. The ERC (European Research Council) Consolidator funded project, “Digital Crossings in Europe: Gender, Diaspora and Belonging” (CONNECTINGEUROPE). Grant Number 647737.

5. For this migration history see Hansen (2003); for background on Turks and the second generation in the Netherlands/Europe see Crul and Vermeulen (2006).

6. Those born in Turkey who re-migrated there from the Netherlands were highest in absolute numbers in 2015 and 2016 Dutch Central Bureau of Statistics (CBS) https://www.cbs.nl/nl-nl/maatwerk/2016/04/migratie-naar-herkomstgroepering-2015. This does not include the second-generation Turkish-Dutch migration to Turkey; the second generation is officially defined as born in the Netherlands.

7. CBS-reported numbers until 2016 http://statline.cbs.nl/Statweb/publication/?DM = SLNL&PA = 60032&D1 = a&D2 = 0&D3 = 0,226&D4 = a&D5 = a&HDR = G4&STB = G1,G2,T,G3&VW = T

8. 2016 CBS report on migration backgrounds of the Dutch population https://www.cbs.nl/nl-nl/achtergrond/2016/47/bevolking-naar-migratieachtergrond

9. Statistics reported by the CBS for years up to 2017 on second-generation migrant population from Turkish background http://statline.cbs.nl/Statweb/publication/?DM = SLNL&PA = 70751ned&D1 = 7&D2 = 235&D3 = a&D4 = a&HDR = T,G3&STB = G1,G2&VW = T

10. CBS report, 2005: most of the second-generation migrant Dutch population intended not to emigrate to the country of their parents’ birth https://www.cbs.nl/nl-nl/nieuws/2005/33/terug-naar-turkije-en-marokko-

11. Klaver, J., Stouten, J., & van der Welle, I. (2010) “Emigratie uit Nederland: een verkennende studie naar de emigratiemotieven van hoger opgeleiden,” report based on research conducted by Regioplan for the Dutch Ministry of Internal Affairs.

12. Translated from Dutch to English by author.

13. Yemeksepeti was designed for Istanbul, specifically. Yandex is available for various cities, and is originally Russian.

14. This is described in somewhat similar ways to Miller’s description of Facebook as a “time suck” (Miller, 2011).

15. As a reference point, Istanbul is the largest city in Europe https://www.worldatlas.com/articles/largest-cities-in-europe-by-population.html Last accessed: 11/05/2019.

16. Here, I am not considering Facebook Messenger’s technical affordance of voice and video calls, because my respondents typically did not use Facebook for calling.

17. Italicized, as it was said in English in a Dutch-language interview.
